# Study on the controllability of the fabrication of single-crystal silicon nanopores/nanoslits with a fast-stop ionic current-monitored TSWE method

**DOI:** 10.1038/s41378-023-00532-0

**Published:** 2023-05-16

**Authors:** Hao Hong, Jiangtao Wei, Xin Lei, Haiyun Chen, Pasqualina M. Sarro, Guoqi Zhang, Zewen Liu

**Affiliations:** 1 Department of Microelectronics, Delft University of Technology, 2628 CD Delft, The Netherlands; 2 School of Integrated Circuits, Tsinghua University, 100084 Beijing, China; 3 School of Chemistry, Beihang University, 100084 Beijing, China; 4 School of Electronic and Information Engineering, Beijing Jiaotong University, 100084 Beijing, China

**Keywords:** Nanopores, Nanopores

## Abstract

The application of single-crystal silicon (SCS) nanopore structures in single-molecule-based analytical devices is an emerging approach for the separation and analysis of nanoparticles. The key challenge is to fabricate individual SCS nanopores with precise sizes in a controllable and reproducible way. This paper introduces a fast-stop ionic current-monitored three-step wet etching (TSWE) method for the controllable fabrication of SCS nanopores. Since the nanopore size has a quantitative relationship with the corresponding ionic current, it can be regulated by controlling the ionic current. Thanks to the precise current-monitored and self-stop system, an array of nanoslits with a feature size of only 3 nm was obtained, which is the smallest size ever reported using the TSWE method. Furthermore, by selecting different current jump ratios, individual nanopores of specific sizes were controllably prepared, and the smallest deviation from the theoretical value was 1.4 nm. DNA translocation measurement results revealed that the prepared SCS nanopores possessed the excellent potential to be applied in biosensing.

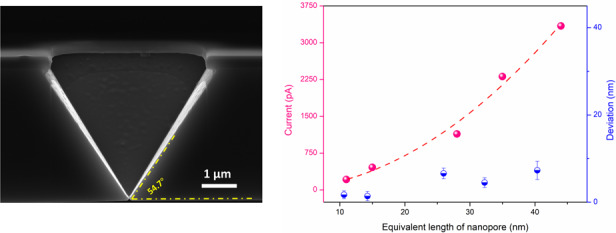

## Introduction

Solid-state nanopores have been widely used to study the fundamentals of ionic and nanofluidic channels^1–3^ to develop detection tools for studying nucleic acids, proteins, and small molecules^4–11^ and to characterize the nanostructure of DNA^12–14^. This is due to the advantages of solid-state nanopores compared with those of biological nanopores in terms of chemical stability, controllable geometry, and process compatibility with existing semiconductor processes and microelectromechanical system (MEMS) techniques. Other applications of solid-state nanopores^15–21^, such as optical modulation^22^, nanopore generators^23^, and molecular separations^24–26^, have also been demonstrated, except for nanopore sensing. Nanopore sensing is mainly based on characterizing the ionic current by applying a transmembrane voltage. The threading of an analyte through the nanopore can cause a temporal blockage current. This change can be correlated to the properties of the analyte, such as the size and surface charge, as well as to the manner of passage, especially for a nonspherically shaped analyte^27–30^. Consequently, narrower and shorter nanopores or nanoslits are better suited for increasing the signal and thereby allow smaller variations in analytes to be resolved^31^. Therefore, special attention has been given to silicon-based nanopores/nanoslits due to their geometric and material characteristics, namely, their short channel length, easy surface modification, and compatibility with semiconductor technology. The conventional methods for obtaining nanopores/nanoslits are mainly divided into dry and wet etching methods. Dry etching methods, such as inductively coupled plasma (ICP), focused ion beam (FIB), and focused electron beam (FEB) drilling, are the most widely used techniques for fabricating nanopores/nanoslits smaller than 10 nm on different thin membranes in a short time^32–34^. Other methods have also been reported, for example, dielectric breakdown is an in situ nanopore fabrication method that uses only a voltage-current reading apparatus, which opens up the possibility of fabricating nanopores integrated with complex, potentially nonplanar, geometries^35–38^. However, there are several challenges in using this technique, such as the formation of multiple nanopores^39,40^. Emmrich et al. reported the FIB fabrication of nanopores with diameters as small as 5 nm^41^. Nevertheless, the low efficiency associated with this method has severely restricted the possibility of large-scale fabrication. The fabrication of nanopores by the abovementioned methods is difficult due to their serial manufacturing characteristics and the fact that they all form cylindrical inner structures, which means that the both spatial and temporal resolution of detection is limited when these nanopores are used in biosensors^42^. Another method that can be used to fabricate hollowed nanostructures on a large scale at a low cost is the TSWE method, which is based on conventional semiconductor processes and MEMS techniques. However, the lack of precise monitoring of the pore-opening event and the rapid fast-stop system has led to severe over-etching, resulting in nanopores that not only are large but also have sizes that are difficult to controllably regulate. To control this process precisely, Wang et al. proposed a color-feedback mechanism based on phenolphthalein mixed with KOH to make the mixed solution turn red to monitor the pore-opening event^43^. However, this method requires a reference pore structure and still presents some challenges concerning pore-opening controllability. Apel and Siwy et al. proposed an ionic current monitoring method to detect the pore-opening process and utilized a stopping medium (acid) to reduce over-etching^44,45^. Park et al. used the same strategy for silicon nanopores and stopped the reaction after the pore opening manually^46^. These methods could improve the precision to some degree. Nevertheless, adding the stopping medium can only slow the etching rate rather than stop the reaction completely; thus, over-etching still occurs. At the same time, the heat released by the neutralization reaction of acid and alkali also causes the acceleration of etching and current fluctuation, which results in difficulties for the current-based controllable preparation of the samples. On the other hand, stopping the reaction manually after pore opening increases over-etching and is not compatible with large-scale manufacturing based on MEMS technology. A method for the controllable fabrication of nanopores based on wet etching is thus highly desirable.

In this work, we report the combination of the TSWE method with a fast-stop highly sensitive ionic current-monitored method for fabricating SCS nanopores/nanoslits with different dimensions. In the third step, pore-opening etching is carefully monitored by the highly sensitive current process, and wet etching is dominated by a fast-stop system. This method dramatically improves fabrication accuracy, precision, and reproducibility and allows a significant reduction in the minimum size that can be achieved. In fact, nanoslits with feature size as small as 3 nm were obtained. By quantitatively analyzing the relationship between current and nanopores and setting different current jump ratios, nanopores of accurate sizes were also obtained. The smallest deviation between the obtained and theoretical values was 1.4 nm. This illustrates that using the proposed fast-stop ionic current-monitored TSWE method, individual SCS nanoholes/slits with accurate dimensions can be efficiently fabricated, enabling large-scale fabrication based on MEMS technology. Finally, the DNA translocation performance of the obtained nanopores was evaluated, and the results indicated excellent possible biosensing applications.

## Experiment

**Fig. 1:**
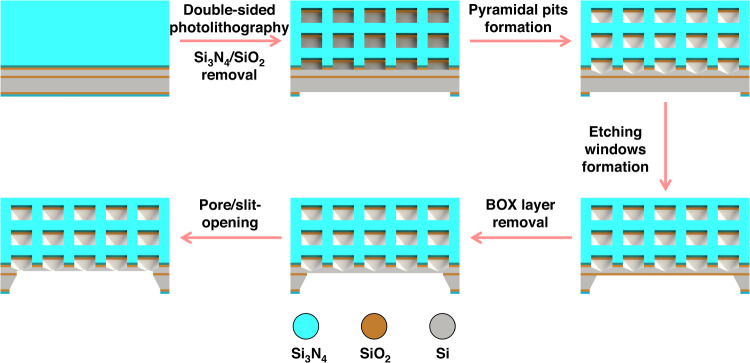
Illustration of the fabrication process. Schematic illustration of the main fabrication process steps of the TSWE method

**Fig. 2:**
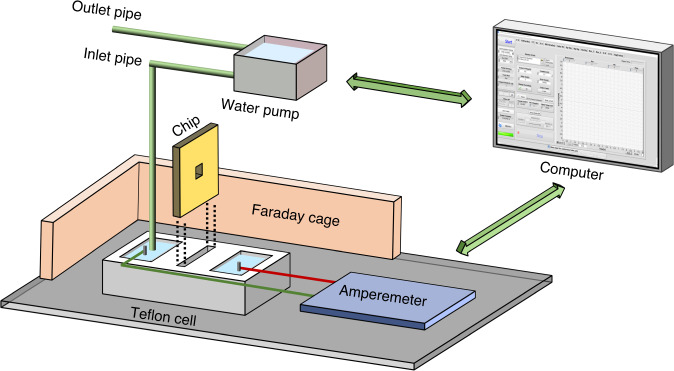
Illustration of the third step of the TSWE method. Schematic illustration of the fast-stop highly sensitive current-monitored etching

A silicon-on-insulator (SOI) wafer (4 inches), composed of a 5.0 ± 0.5 μm-thick undoped silicon (100) device top layer, which was separated from the 300 ± 10 μm undoped bulk silicon (100) substrate by a 1000 ± 50 nm buried oxide layer (SiO_2_, “BOX”), was used as the initial substrate. Figure 1 depicts the main fabrication process of the proposed TSWE method. First, 100 nm SiO_2_ and 200 nm Si_3_N_4_ films were grown on both sides of the cleaned SOI wafer by thermal oxidation and low-pressure chemical vapor deposition (LPCVD) as a stress buffer and mask layer, respectively. The patterns were transferred to both sides of the wafer by double-sided photolithography, and ICP was utilized to remove the Si_3_N_4_ and SiO_2_ layers to open the etching windows on both sides of the wafer. Next, the first step of the TWSE method was carried out, namely, wafer-scale etching on the front side of the wafer in a 33 wt% KOH solution at 80 °C, and pyramid-shaped pits were formed. As the second step of the TWSE method, the same process was performed to produce the back etching windows, and the BOX layer was removed by buffered hydrofluoric acid. After that, a silicon film of ~1.5 μm was exposed, which was etched away to open the nanopore/slit during the third step. For the third step of the TSWE method, a fast-stop highly sensitive current-monitored etching experimental setup was developed to detect the pore-opening event, as shown in Fig. 2. There were two chambers (made in Teflon) separated by a silicon chip: one contained KCL (1 M) solution (front side) and the other contained 33 wt% KOH solution (backside). Ag/AgCl electrodes connected to the measuring unit (Keithley 2450) were placed in both chambers and used to detect changes in current. A pipe for pumping the solution was placed in the chamber. The whole apparatus was placed into a Faraday cage during the measurement to weaken the environmental interference. Once the nanopore/nanoslit was opened (~45 min was required to open the nanopore/slit), the current reached the set current jump ratio (the ratio of the current after the pore opening to the background current before the pore opening), and the connected computer simultaneously triggered the fast-stop system and drove the water pump (flow rate ~60 mL/min) to pump out the etching solution (~0.2 mL), and nanopores or nanoslits with a precise area were obtained.

## Results and discussion

**Fig. 3:**
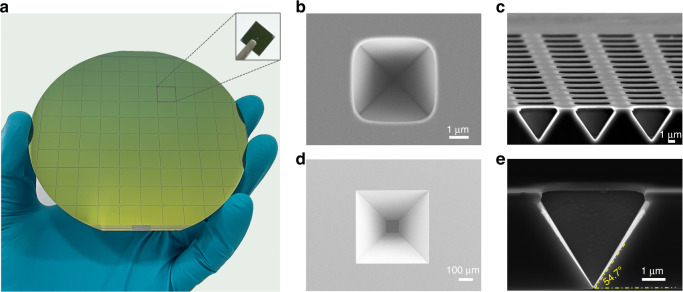
Photograph and SEM images of the chip during fabricaiton. **a** Photograph of a wafer after the first step. **b** SEM micrograph of a pyramid pit. **c** Cross-sectional SEM image of a pyramid pit array. **d** SEM micrograph of the back etching window. **e** Cross-sectional SEM image of an individual nanopore with a size of 15 nm

Figure 3a shows a photograph of the wafer (with its front side), which contains 88 chips of size 9 × 9 mm, as shown in the inset image. Here, we use three kinds of mask patterns to prepare different nanostructures, as described in Table 1. Figure 3b shows a scanning electron microscope (SEM) micrograph of a pyramid pit, which is obtained after the first step of wet etching in a chip with an individual nanopore. Rounded corners in the hard-mask layer are observed. Figure 3c exhibits a cross-sectional SEM image of a pyramid pit array, showing good coincidence, and the morphology of the pits conforms to anisotropic etching theory. In fact, considerable geometric diversity is observed after careful measurements. After the second step of the TSWE method, on the backside of the chip, a larger wet etching window than that on the front side is opened. The bottom size of the window is 87.7 × 89.9 µm^2^, and this area contains an array of individual pre-etched pyramidal pits on the other side of the chip. Finally, Fig. 3e shows a cross-sectional SEM image of an individual nanopore with a size of 15 nm. The pyramidal structure with an angle of 54.7° is preserved. The anisotropic wet etching results in undercutting under the hard mask layer, even though the etching rate ratio of the (111) crystal plane to (100) is as high as 1/50 at room temperature with 33 wt% KOH.

**Table 1:** Three types of front-side mask patterns

Mask type	Number (in one chip)	Size
Nanoslit array	16*16	4 × 4.5 µm
Nanopore array	16*16	4 × 4 µm
Individual nanopore	1	4 × 4 µm

**Fig. 4:**
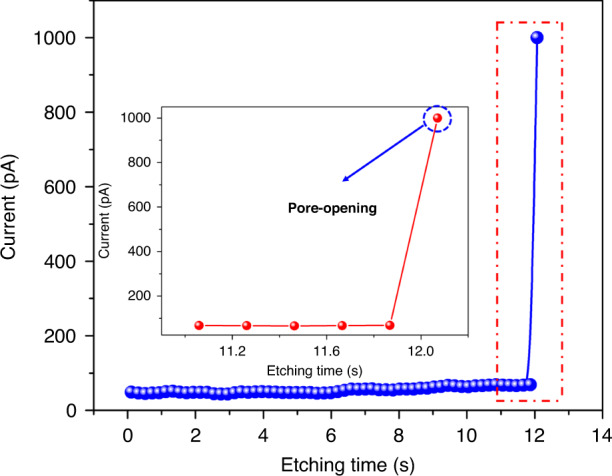
Current curve during the pore-opening. Current–time curves recorded during the slit-opening event

**Fig. 5:**
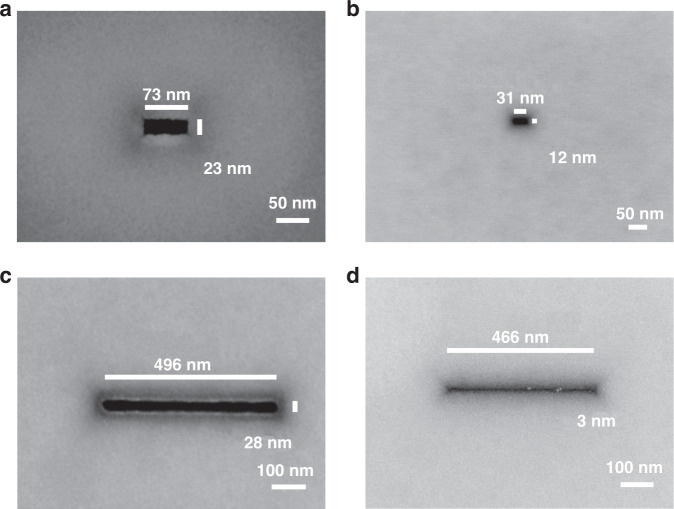
SEM images of the obtained nanopores and nanoslits. SEM micrographs of nanopores **a** 23 nm, **b** 12 nm, and nanoslits **c** 28 nm, and **d** 3 nm fabricated by the TSWE method

Controlling over-etching is always a major challenge in fabricating nanopores with extremely small sizes. Improving the monitoring sensitivity of the pore-opening event and utilizing a fast-stop system can effectively reduce over-etching and guarantee a small size. During the experiment, a large zero-bias current spontaneously flows through the silicon chip as soon as the chip is brought into contact with the two electrolytes (KCl and KOH solution). This is due to electrons that are generated at the interface between Si and the KOH solution during the etching process. By applying a 0.8 V bias voltage between the two Ag/AgCl electrodes, the electrochemical potential difference at the KCl/Si interface and the KOH/Si interface is counterbalanced, thereby minimizing the zero-bias current. Here, we use the chips of nanopores and nanoslit array masks to fabricate nanopores and nanoslits. Figure 4 shows the variation in current based on the fast-stop ionic current-monitored method during the pore-opening event. Before the pore-opening event, the background current is stable and is ~50 pA. Once the pore-opening event takes place, the current immediately increases and reaches the set current jump ratio of 20 (current after pore-opening ~1000 pA). This is caused by the exchange of electrolyte solutions on both sides of the chip once the nanopores or nanoslits are opened. Obviously, the mechanism fails if the current jump ratio setting is small due to possible noise current pulses. On the other hand, a large current jump ratio delays the feedback of the pore-opening event, increasing the over-etching time. Therefore, to controllably fabricate nanopores or nanoslits with specific sizes, setting a precise current jump ratio is an extremely critical step. With the reported fast-stop highly sensitive current-monitored TSWE method, nanopores and nanoslits with nanoscale features are successfully realized. Figure 5 shows the SEM images of the obtained nanopores with feature sizes of 23 and 12 nm. It should be noted that for most obtained nanopores, the shape of the as-etched nanopore is a rectangle rather than a square due to the imperfect hard mask and photolithography. Nanoslits with feature sizes of 28 and 3 nm are also obtained. To the best of our knowledge, this is the smallest feature size (3 nm) of a nanoslit obtained by the TSWE method. It is worth noting that the slit has a length of 500 nm, which is controlled by the size of the front pyramid pit. The pictures clearly show the SCS nanopore/slit fabrication capability of the proposed method and that it is possible to manufacture nanopores/slits with extremely small feature sizes.

**Fig. 6:**
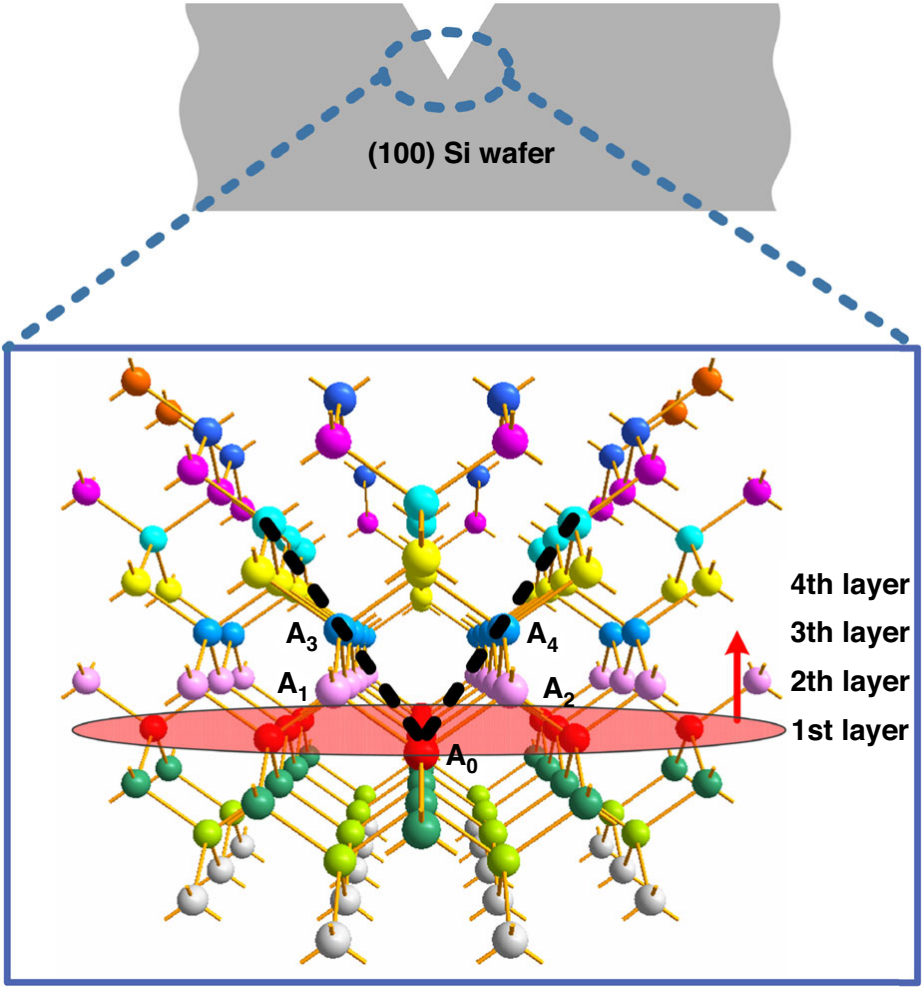
Etching front progression from an atomic perspective. Schematic diagram of the arrangement of silicon atoms and the etching front progression during etching

To clarify the theoretical minimum value of the nanopore feature size obtained by wet etching, we performed an analysis on the SCS atomic layers. According to the anisotropic etching characteristics of silicon, after the first step of wet etching, an inverted pyramid structure composed of four (111) planes is formed and intersects a silicon atom on a (100) plane. To simplify the analysis, we assume that the silicon atoms are etched layer by layer along the (100) direction during the third step. Under this assumption, we constructed a schematic diagram of the arrangement of silicon atoms, as shown in Fig. 6, in which each silicon atom is connected by chemical bonds with two silicon atoms in each of the two adjacent layers. The silicon atoms of the same color are arranged on the same (100) plane, and the silicon atoms on the black dotted line are located on the same (111) plane. As shown in Fig. 6, the two black dotted lines intersect at silicon atom A_0_ on the 1st layer. During the third step of the etching, the silicon atoms are etched layer by layer along the direction of the arrow. When the silicon atom A_0_ of the 1st layer is etched away, the A_1_ and A_2_ atoms are exposed and remain because these two atoms are connected by chemical bonds with the silicon atoms A_3_ and A_4_ of the 3rd layer. At this time, it can be considered that the nanopore is formed, and the nanopore obtained currently is within its theoretical minimum size, which is the distance between two adjacent silicon atoms on the (110) plane, as $\sqrt {{{2}}} {{{a}}}/{{{2}}}$ (*a* is the lattice constant of silicon, 0.54 nm). Therefore, the theoretical minimum feature size of the nanopores obtained by wet etching is 0.38 nm. Due to over-etching, the silicon atomic layer continues to be etched to form larger nanopores. It can be concluded that when silicon atoms on the *n*th layer are etched away, the feature size of the nanopore is $\frac{{\sqrt {{{2}}} {{{a}}}}}{{{{2}}}} \cdot \left\lceil {\frac{{{{n}}}}{{{{2}}}}} \right\rceil$. Therefore, since the nanopores are just formed and then continue to increase in size, their feature sizes should be 0.38, 0.76, 1.14 nm, and so on. Thus, it can be understood that the nanoslit exhibited in Fig. 5d is the result obtained after the 15th or 16th layer of silicon atoms is etched, and its theoretical feature size should be 3.04 nm. These results reveal the potential of the proposed TSWE method for fabricating nanopores/nanoslits with extremely small feature sizes.

To controllably fabricate individual nanopores with accurate dimensions by the proposed current-monitored TSWE method, the relationship between the size of the nanopore and the current must be understood. According to the reports of Dekker et al.^31,47^, this relationship can be described by Eq. (1):

1 $${{{i}}}{{{ = }}}{{{V}}}\frac{{{{{d}}}_{{{{{\rm {pore}}}}}}^{{{2}}}}}{{{{{L}}}_{{{{{\rm {eff}}}}}}}}\left[ {\left( {{{{\mu }}}_{{{{\rm {k}}}}}{{{ + }}}{{{\mu }}}_{{{{{\rm {Cl}}}}}}} \right){{{n}}}_{{{{{\rm {kCl}}}}}}{{{e}}}{{{ + }}}{{{\mu }}}_{{{{\rm {k}}}}}\frac{{{{{4}}}{{{\sigma }}}}}{{{{{d}}}_{{{{{\rm {pore}}}}}}}}} \right]$$

Here, *d*_pore_ is the equivalent length ($\sqrt {{{{\rm{area}}}}}$) of the nanopore/nanoslit. It should be noted that for most obtained nanopores, the shape of the as-etched nanopore is a rectangle rather than a square, as indicated by the initial lithographic pattern. This is caused by imperfections in the mask and the photolithography process, so here, we use the equivalent length to facilitate the analysis (rectangular nanopore length equivalent to the square nanopore length, ${{{ab}}} = {{{d}}}_{{{{\rm{pore}}}}}^{{{2}}}$). *V* is the bias voltage between the two electrodes. *n*_kCl_ is the number density of potassium or chloride ions, *e* is the elementary charge, and *μ*_k_ and *μ*_Cl_ are the electrophoretic mobilities of potassium and chloride ions, respectively, while *σ* is the surface charge density. The first term in Eq. (1) represents the current contributed by the bulk conductance, and the surface charge conductance contribution to the current in the nanopore is represented by the second term. We can use it to analyze the relationship between nanopore sizes and the current of two nanopores as follows:

2 $$\frac{{{{{i}}}_{{\rm {exp}}}}}{{{{{i}}}_{{\rm {ref}}}}} = \frac{{{{{V}}}\frac{{{{{d}}}_{{\rm {pore}}_{{\rm {exp}}}}^2}}{{L_{{\rm {eff}}}}}\left[ {\left( {{{{\mu }}}_{{{\rm{k}}}} + {{{\mu }}}_{\rm {{C}}{{{{{l}}}}}}} \right)n_{{{{{\rm {k}}}}}{\rm{C}{{{{l}}}}}}e + {{{\mu }}}_{{{\rm{k}}}}\frac{{4{{{\sigma }}}}}{{{{{d}}}_{{\rm{pore}}_{\rm{exp}}}}}} \right]}}{{{{{V}}}\frac{{{{{d}}}_{{\rm{pore}}_{\rm{ref}}}^2}}{{L_{{\rm {eff}}}}}\left[ {\left( {{{{\mu }}}_{{{\rm{k}}}} + {{{\mu }}}_{{\rm {C}}{{{l}}}}} \right)n_{{{{{\rm {k}}}}}{\rm {C}{{{{l}}}}}}e + {{{\mu }}}_{{{\rm{k}}}}\frac{{4{{{\sigma }}}}}{{{{{d}}}_{{\rm{pore}}_{\rm{ref}}}}}} \right]}}$$

**Fig. 7:**
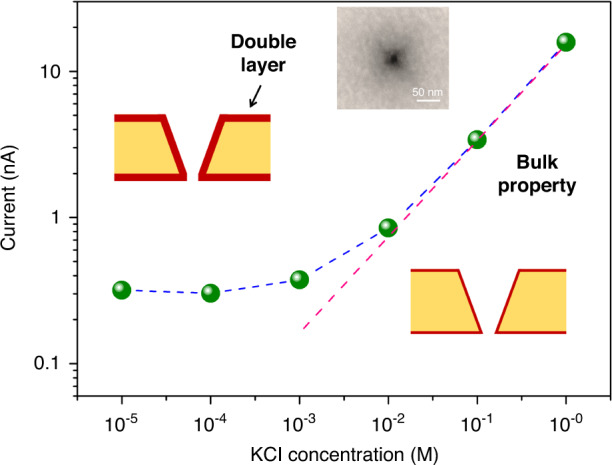
Electrical characterization of the nanopore. Relationship between the ionic current and KCl electrolyte concentration

Here, *i*_exp_ and *i*_ref_ represent the current of an expected nanopore and a reference nanopore, respectively. To simplify Eq. (2), we measure the ionic current through a nanopore (~18 nm) for different KCl electrolyte concentrations. In the high concentration region (Debye length ≤ nanopore diameter), the ionic current is linearly dependent on the electrolyte concentration, which is indicative of bulk behaviors and properties, as shown in Fig. 7. However, as the concentration decreases, the current deviates from the bulk properties near 10^–3^ M and saturates to yield another slope, which corresponds to a surface charge-governed region (<10^–3^ M). The inset images schematically depict the formation of electrical double layers (red color region) consisting of counterions that screen the surface charge. At low concentrations, a long-range screening region allows the nanopores to be dominantly filled with counterions by overlapping the double layer, which causes the saturation of the ionic current. It could be demonstrated that for the high-concentration (1 M) KCl solution, the current contributed by the bulk conductance dominates, which means that the first term in Eq. (1) dominates the current^48^. Thus, Eq. (2) can be simplified as follows:

3 $$\frac{{{{{i}}}_{{\rm {exp}}}}}{{{{{i}}}_{{\rm {ref}}}}} = \frac{{{{{V}}}\frac{{{{{d}}}_{{\rm {pore}}_{{\rm {exp}}}}^2}}{{L_{{\rm {eff}}}}}\left[ {\left( {{{{\mu }}}_{{{\rm{k}}}} + {{{\boldsymbol{\mu }}}}_{{\rm {C}}{{{\rm{l}}}}}} \right)n_{{{{\rm{k}}}}{\rm {C}}{{{{l}}}}}e} \right]}}{{{{{V}}}\frac{{{{{d}}}_{{\rm{pore}}_{\rm{ref}}}^2}}{{L_{{\rm {eff}}}}}\left[ {\left( {{{{\mu }}}_{{{\rm{k}}}} + {{{\mu }}}_{{\rm {C}}{{{\rm{l}}}}}} \right)n_{{{{\rm{k}}}}{\rm {C}}{{{\rm{l}}}}}e} \right]}} = \frac{{{{{d}}}_{{\rm {pore}{\rm{exp}}}}^2}}{{{{{d}}}_{ {\rm{pore}{\rm{ref}}}}^2}} = \frac{{{{{A}}}_{{\rm {exp}}}}}{{{{{A}}}_{{\rm {ref}}}}}$$

Here, *A* is the area of the nanopore, and *d*_pore_ is the equivalent length ($\sqrt {{{A}}}$) of the nanopore/nanoslit. According to Eq. (3), once a nanopore with a specific area and the corresponding current are defined, then an appropriate current jump ratio can be set according to the current to obtain expected nanopores with a specific area to achieve the controllable preparation of nanopores. Consequently, we set different current jump ratios and obtain different nanopores with equivalent lengths.

**Fig. 8:**
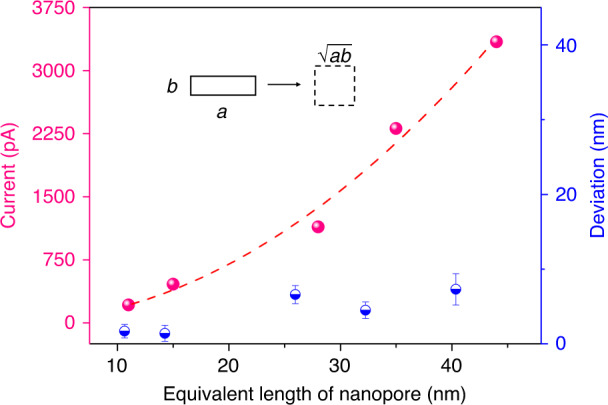
Analysis of the controllable fabrication method for nanopores. Relationship between the size of individual nanopores and the ionic current and the deviation between the equivalent length of the obtained nanopores and the theoretical value

From Eq. (3), it appears that the current of two different nanopores is proportional to the area. Here, we use an individual nanopore (obtained under the same experimental conditions) of a known area and corresponding current (area of 846 nm^2^ and current of 2100 pA) as a reference. By setting the current jump ratio to 5, 10, 20, 40 and 80 (background current is ~50 pA), individual nanopores with equivalent lengths ($\sqrt {{{{ab}}}}$) of 11, 15, 28, 35, and 44 nm are obtained, and the related ionic currents are also recorded, as shown in Fig. 8. The fitting curve demonstrates that the relationship between the equivalent length of the nanopores and the current follows Eq. (3), where the fitting equation is *y* = 1.74*x^2^ and the fitting *R*-squared value is 0.99. Then, we use the obtained five currents shown in Fig. 8 to calculate the corresponding theoretical length of the five nanopores. The theoretical equivalent lengths of the five nanopores are 9.3, 13.6, 21.4, 30.5, and 36.7 nm, and the deviations between the obtained and theoretical lengths are 1.7, 1.4, 6.6, 4.5, and 7.3 nm, respectively, as shown in Fig. 8. The actual length of all the obtained nanopores is larger than the theoretical value because there is still <0.2 s of over-etching time after triggering the fast-stop system, which pumps out the etching solution to stop the reaction. In addition, we find that the error increases as the length of the nanopore increases. Different from the anisotropic etching properties before pore opening, in which the etching is mainly dominated by the (100) plane, the (100), (110) and (111) planes apparently contribute to the expansion of the nanopore after pore opening with an etching rate of approximately 3 nm/s. In fact, more planes, such as (210), (211), (310), and (311), are also simultaneously etched after pore opening, resulting in an acceleration of the expansion of the nanopore area. Therefore, a longer over-etching time causes a more significant error. It is predictable that when the jump ratio is as small as 1, the corresponding current of an expected nanopore is only approximately 50 pA. According to the above theory, we can controllably obtain an individual nanopore with an area of ~20.1 nm^2^, with an equivalent length of only 4.5 nm. While maintaining the area under the same value, the shape (length–width ratio) of the nanopore can be easily tuned by changing the layout of the wet etching mask^49^. If the background current can be further reduced, the size of the controlled nanopore can be further reduced. Here, we demonstrated that the proposed TSWE method has the potential to fabricate extremely small dimensions, which can be adjusted by tuning the aspect ratio of the mask to produce nanopores with extremely small feature sizes. Moreover, a comparison of the main fabrication methods of solid-state nanopores is shown in Table 2. It can be concluded that the proposed TSWE method provides an effective strategy for controllably realizing extremely small silicon-based nanopores/slits and could be used in large-scale MEMS fabrication at a low cost.

**Table 2:** Comparison of the main fabrication methods of solid-state nanopores

Method	Inner morphology	Fabrication scale	Manufacturing cost	Pore size (nm)	Refs.
FIB/FEB	Cylindrical/hourglass	One at a time	High	>1.8	^41,50–53^
Dielectric breakdown	Cylindrical	One/multiple at a time	Low	>1.1	^36–39,54–56^
Nanoimprinting	Conical	Single/array	Low	>6	^57,58^
Metal-assisted etching	Conical	Single/array	Middle	>20	^59–61^
TSWE	Pyramidal	Single/array	Low	>3	This work

**Fig. 9:**
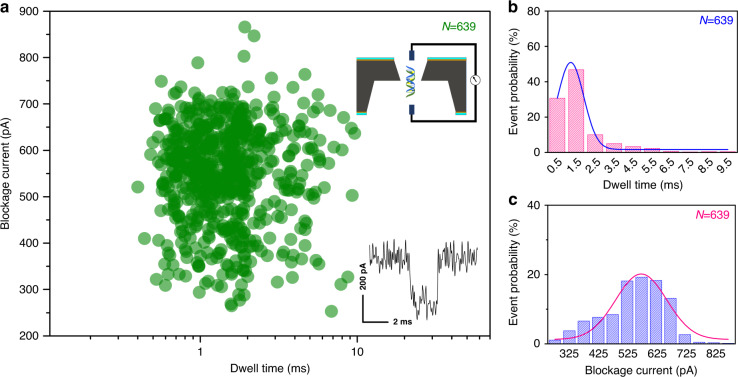
DNA translocation measurement. DNA translocations through a nanopore with a feature size of 11.3 nm. **a** Corresponding event density plot constructed from 639 translocation events at an applied bias of 0.4 V, **b** dwell time, and **c** blockage current histogram

To demonstrate that nanopores with pyramid structures have excellent potential for biosensing tests were carried out. The blockage current during DNA translocation events was measured by a patch-clamp amplifier (Axopatch 200B, Axon Instruments, USA). The signal data were sampled at a rate of 500 kHz and low-pass filtered at 10 kHz. Then, the analog signals were converted into digital data by a data-acquisition module (Digidata 1440A, Axon Instruments, USA). The entire experiment was carried out on a vibration isolation table. Figure 9 shows the dwell time and blockage current distribution of λ-DNA (48.5 kbp, 1 ng/μL) passing through a pyramidal nanopore with a feature size of 11 nm under 0.4 V. The nanopore was filled with a buffer electrolyte of 1 M KCl, 10 mM Tris, and 1 mM ethylenediaminetetraacetic acid (EDTA) at pH = 8.0, and the current-time curves were measured between two Ag/AgCl electrodes placed across the SCS nanopore. It is evident from the results shown in Fig. 9a that the dwell time and blockage current of DNA molecules passing through the nanopore is concentrated in the dark area of the scattergram. The blockage current and dwell time shift is partially due to the symmetrical folding, breaks, and overlaps of a portion of the DNA. The inset figure shows more details about the typical ionic current signal. Figure 9b and c show the dwell time and blockage current distributions corresponding to Fig. 9a, respectively. The average blockage current is calculated to be 548.9 pA, and the transit time is calculated to be 1.77 ms. From this, we know that the DNA passing rate is 27.4 kbp/ms. It should be noted that many studies have confirmed that DNA movement in nanopores can be slowed by electrically modulating the wall surface charge based on a careful analysis of the induced electroosmotic flow and DNA−nanopore electrostatic interactions^62–64^, and the nanopores prepared in this report can also reduce the bias rate of DNA molecules by applying gate voltage^65^, which may indicate that the obtained SCS nanopores have good potential for biosensing.

## Conclusion

SCS nanopores/nanoslits with different sizes were obtained by the fast-stop ionic current-monitored TSWE method. With the well-controlled experimental procedure described in this report, the accuracy of fabrication was improved effectively, and extremely small nanoslits (down to 3 nm) were obtained. According to the relationship between the ionic current and the size of nanopores, different current jump ratios were selected to produce nanopores with a specific size. Compared with the theoretical size, the smallest deviation was 1.4 nm, which indicates that this method is an effective method for the controllable fabrication of nanopores/nanoslits with specific sizes. In addition, we conclude that the proposed method can be used to controllably fabricate individual nanopores with an equivalent size of ~4.5 nm and is expected to allow the preparation of nanopores of smaller sizes through the reduction of the zero-bias current. Finally, the biosensing performance of the obtained pyramid nanopores was verified. Overall, the fast-stop highly sensitive ionic current feedback-monitored TSWE method provides a new approach for the controllable and repeatable fabrication of a variety of nanostructures.
