# Correction: Study on the controllability of the fabrication of single-crystal silicon nanopores/nanoslits with a fast-stop ionic current-monitored TSWE method

**DOI:** 10.1038/s41378-023-00564-6

**Published:** 2023-07-25

**Authors:** Hao Hong, Jiangtao Wei, Xin Lei, Haiyun Chen, Pasqualina M. Sarro, Guoqi Zhang, Zewen Liu

**Affiliations:** 1 Department of Microelectronics, Delft University of Technology, 2628 CD Delft, The Netherlands; 2 School of Integrated Circuits, Tsinghua University, 100084 Beijing, China; 3 School of Chemistry, Beihang University, 100084 Beijing, China; 4 School of Electronic and Information Engineering, Beijing Jiaotong University, 100084 Beijing, China

**Keywords:** Nanopores, Nanopores

Correction to: *Microsystems & Nanoengineering*

10.1038/s41378-023-00532-0 published online 16 May 2023

Correction Following publication of the original article^1^, it was noticed that the phrase ‘DNA sequencing’ is incorrect, which should be replaced by ‘biosensing’.

The original paper has been updated.
